# Supplementary material for: Endophyte Bacillus subtilis evade plant defense by producing lantibiotic subtilomycin to mask self-produced flagellin
Source: Commun Biol. 2019 Oct 10;2:368. doi: 10.1038/s42003-019-0614-0 (PMC6787100; doi:10.1038/s42003-019-0614-0)
Supplement: Supplementary file 1 — Supplementary Information [file 42003_2019_614_MOESM1_ESM.pdf]

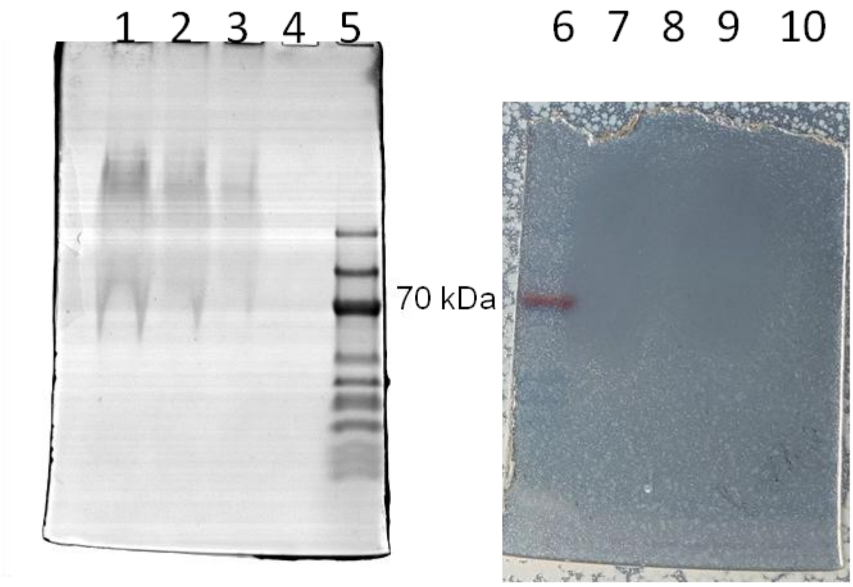

Supplementary Fig. 1 A full and uncropped presentation for Fig. 1b. Lane 1-3 refers to Apn5 proteins with the loading amount of 20 μL, 10 μL, and 5 μL, respectively. Lane 4 is a negative control with the loading amount of 10 μL ddH_2O. Lane 5 refers to Protein marker 26616 (Thermo Scientific™). Lane 6 is protein marker 26616 (Thermo Scientific™). Lane 7 and lane 9 present the Apn5 proteins with the loading amount of 10 μL and 5 μL, respectively. Lane 8 and lane 10 present the negative control with the loading amount of 10 μL ddH_2O.. After electrophoresis, the gel contained lane 1-5 was transferred for CBB strain, and the gel contained lane 6-10 was transferred on an indicator agar plate for inhibition assay. The indicator strain is CU1065.

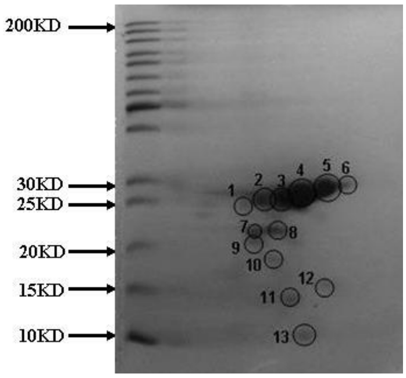

Supplementary Fig. 2 The assay of the antibacterial protein Apn5 by 2D SDS-PAGE. Numbered spots were collected for further MS identification and listed in Supplementary Table 1.

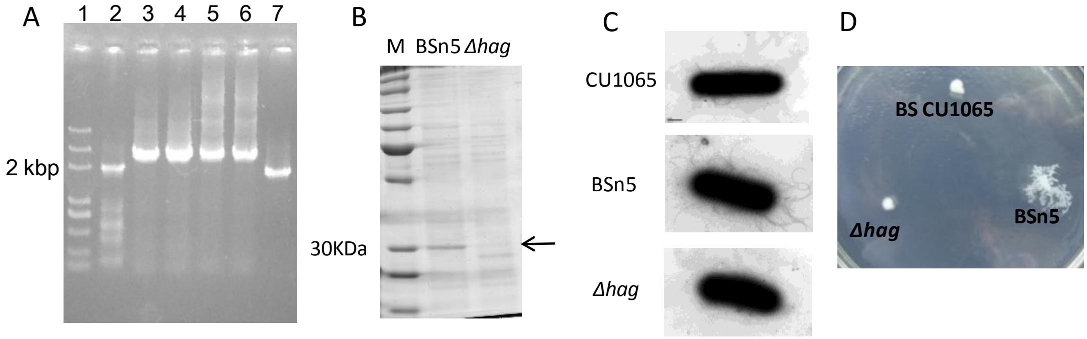

Supplementary Fig. 3 The phenotypes of recombination strain Δhag. (A) Colony PCR verification of mutant Δhag by amplifying gene *hag*. Lane (1) Marker 2k plus. PCR amplys gene *hag* using templates of the BSn5 total DNA (2) and DNA extracts from different transformants (3-7). (B) SDS-PAGE assay on the total proteins of BSn5 and mutant Δhag. (C) Scanning electron microscope verification of mutant Δhag, BSn5 and *B. subtilis* strain CU1065. (D) Mobility assay of mutant Δhag, BSn5 and CU1065.

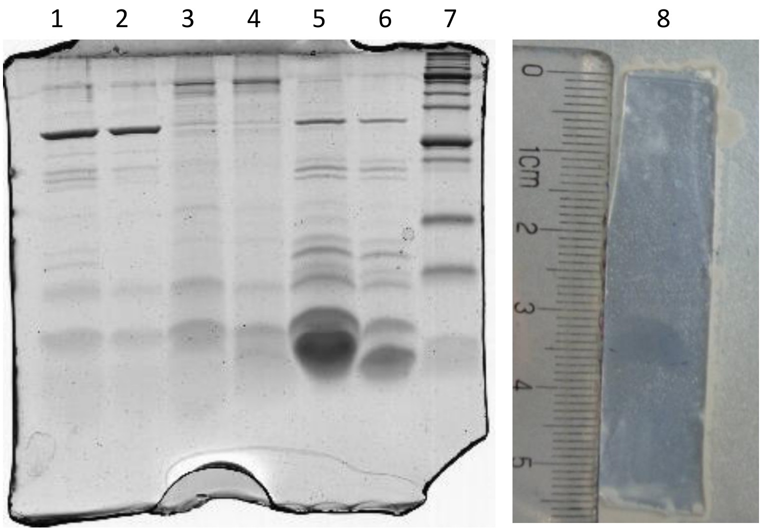

Supplementary Fig. 4 A full and uncropped presentation for Fig. 1d. Lane 1-2 refers to protein Apn5. Lane 3-4 refers to an analugue sample of Apn5 from mutant strain hag. Lane 5-6 refers to the Apn5 protein from the different batch that saved longer. Lane 7 refers to protein Marker 26632 (Thermo Scientific™). Lane 8 refers to the parallel replicates of Lane 3. After electrophoresis, the gel contained lane 1-7 was transferred for CBB strain. The gel contained lane 8 was transferred on an indicator agar plate for inhibition assay.

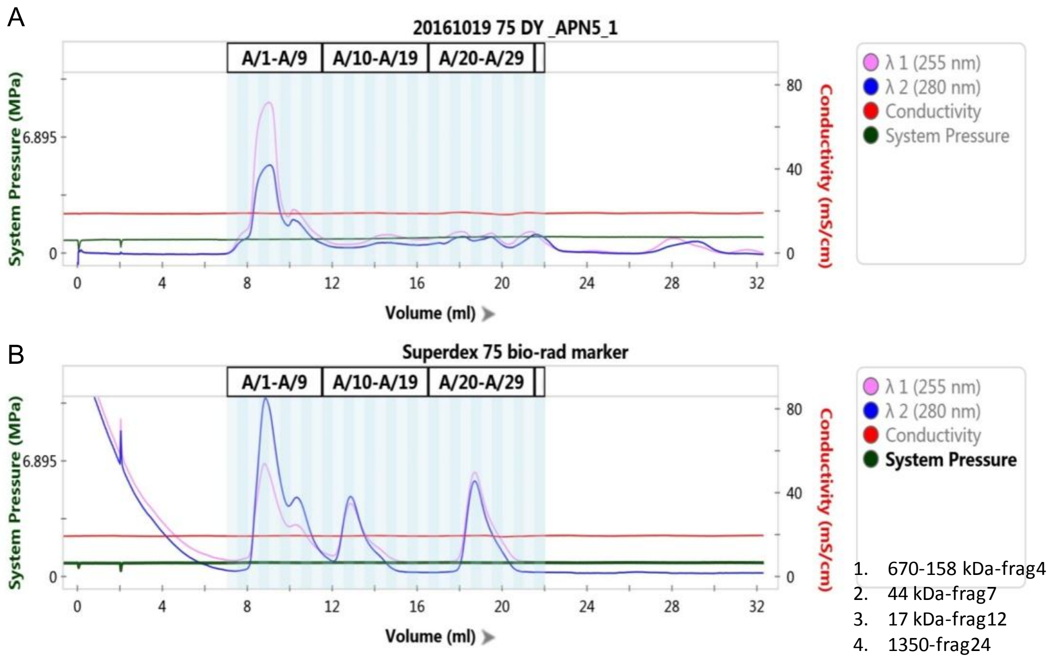

Supplementary Fig. 5 Analysis of the inhibition protein Apn5 (A) and Gel Filtration Standard (Bio-rad) components with molecular weight of (1) 670 kDa, 158 kDa, (2) 44 kDa, (3) 17k Da, and (4) 1350 Da by size exclusion chromatograms (B). Two replicates were performed with consistent result.

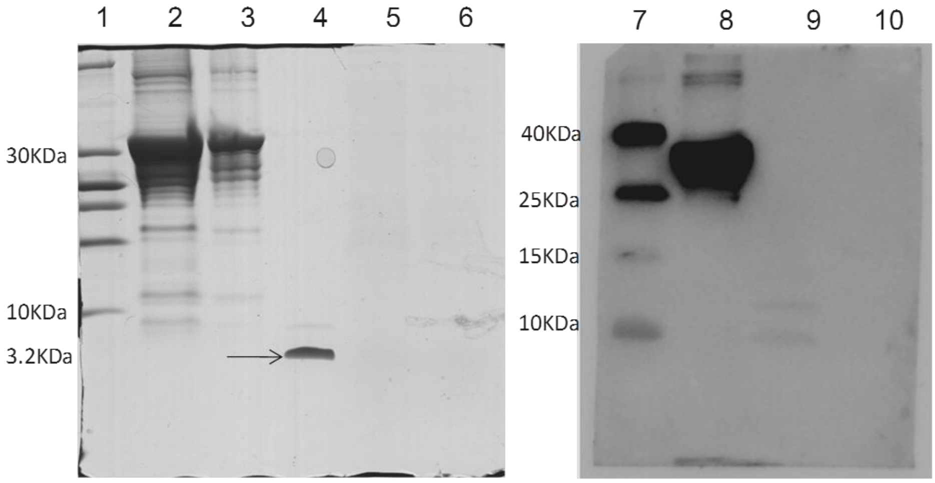

Supplementary Fig. 6 A full and uncropped presentation for Fig. 1f. Lane 1 refers to Protein Marker 26632. Lane 2 refers to His tagged Hag with a loading amount of 10 μL. Lane 3 refers to His tagged Hag with a loading amount of 5 μL. Lane 4 refers to purified subtilomycin. Lane 5-6 refers to negative control by running loading buffer. Lane 7 is a prestained protein Marker ((Thermo Scientific™). Lane 8-10 refers to parallel replicates for Lane 3-5 respectively. The gel contained lane 1-6 was transferred for CBB strain. The gel contained lane 7-10 was transferred on nitrocellulose membrane for ligand blot assay.

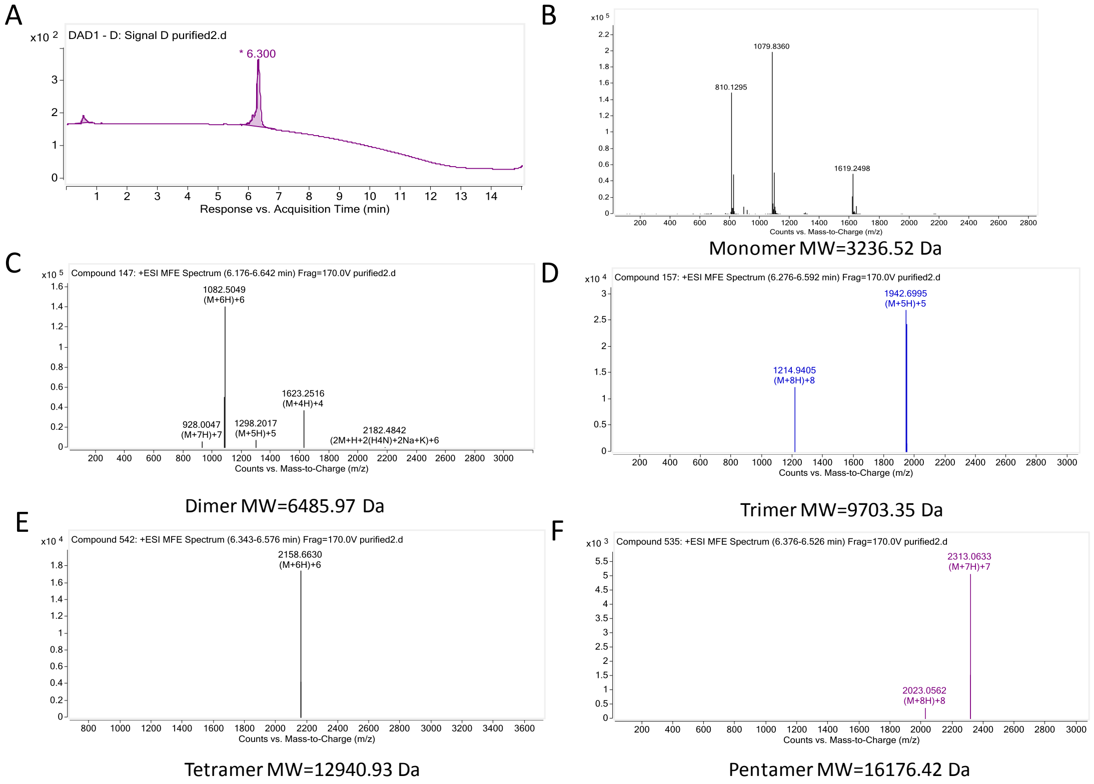

Supplementary Fig. 7 Detection of the polymers of subtilomycin by LC-MS. (A) Liquid chromatography analysis of purified subtilomycin. The detection was using the wavelength 230 nm. The mass spectra respectively showed the monomer (B), dimer (C), trimer (D), tetramer (E), and pentamer (F) of subtilomycin from LC-MS analysis.

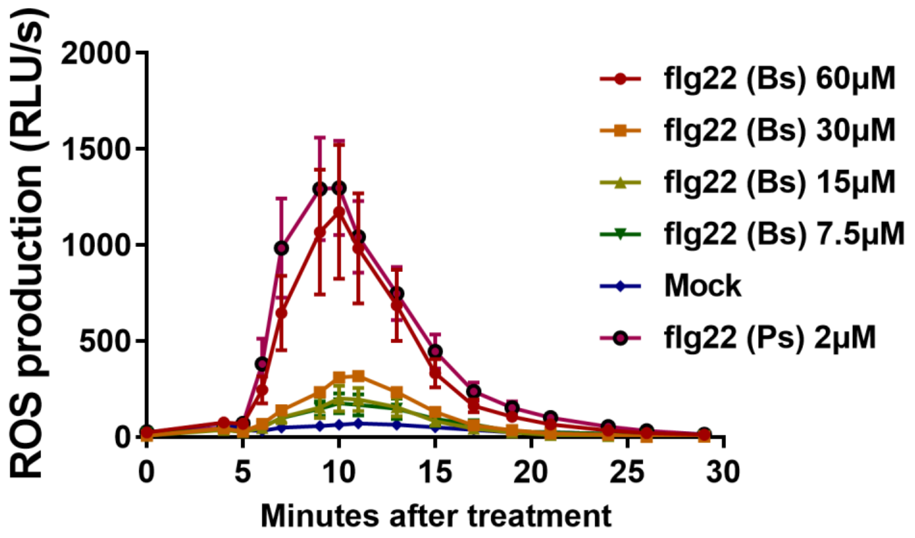

Supplementary Fig. 8 The ROS induced by the 2 μM flg22 peptide from *Pseudomonas syringae*, flg22 (Ps), and the gradient concentrations (7.5~60 μM) of flg22 peptide from *B. subtilis*, flg22 (Bs). Standard errors were from six repeats.

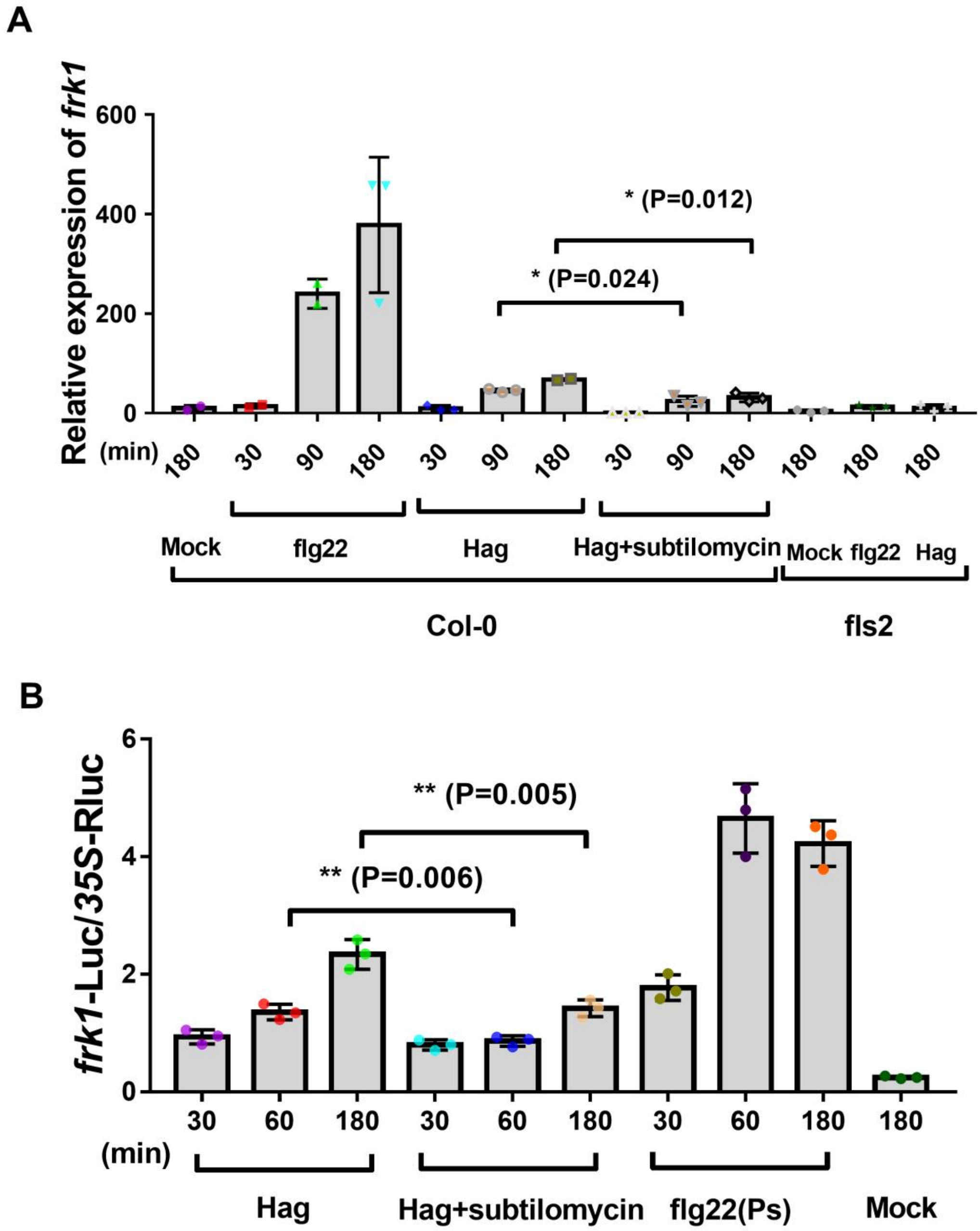

Supplementary Fig. 9 Inhibition of Hag-induced *frk1* gene expression by subtilomycin in *A. thaliana* Col-0 and mutant *fls2-1*. Gradient induction time 30 min, 90 min, and 180 min were set for RT-qPCR assay (A) 2-3 technical repeats were applied. "*"indicates significant difference (P<0.05) between Hag and Hag with subtilomycin for 90 min and 180 min, respectively. (P value=0.024 and 0.012 respectively). (B) Inhibition of Hag-induced *frk1::LUC* activity by subtilomycin in wild type *A. thaliana* Col-0 leaf protoplasts using dual reporter system. Gradient induction time 30 min, 90 min, and 180 min were set for duel luciferase reporter assay. Repeats were from three measurements. "**"indicates significant difference (P<0.01) between Hag and Hag with subtilomycin for 90 min and 180 min, respectively. The applied concentration of Hag and subtilomycin is 30 μM and 15 μM, respectively.

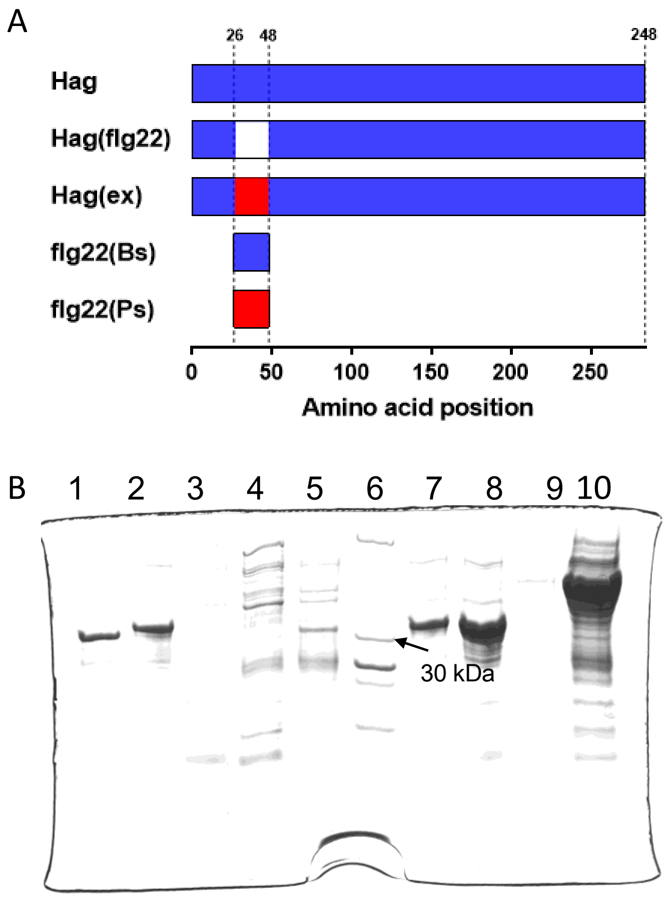

Supplementary Fig. 10 Construction of the truncated and recombinant Hag proteins. (A) Diagram indicates the design of the truncated and recombinant Hag proteins. (B) Tricine-SDS-PAGE analysis of the purification of the proteins from *E. coli*, Lane (1) ΔHag, (2) Hag, (6) marker 26632 (Thermo Scientific™), (7) Hag (ex) (9) Loading buffer and (10) FliC (Se). The other lanes were not related to this test. The amount of 10 μL for each sample was loaded in each well for assay.

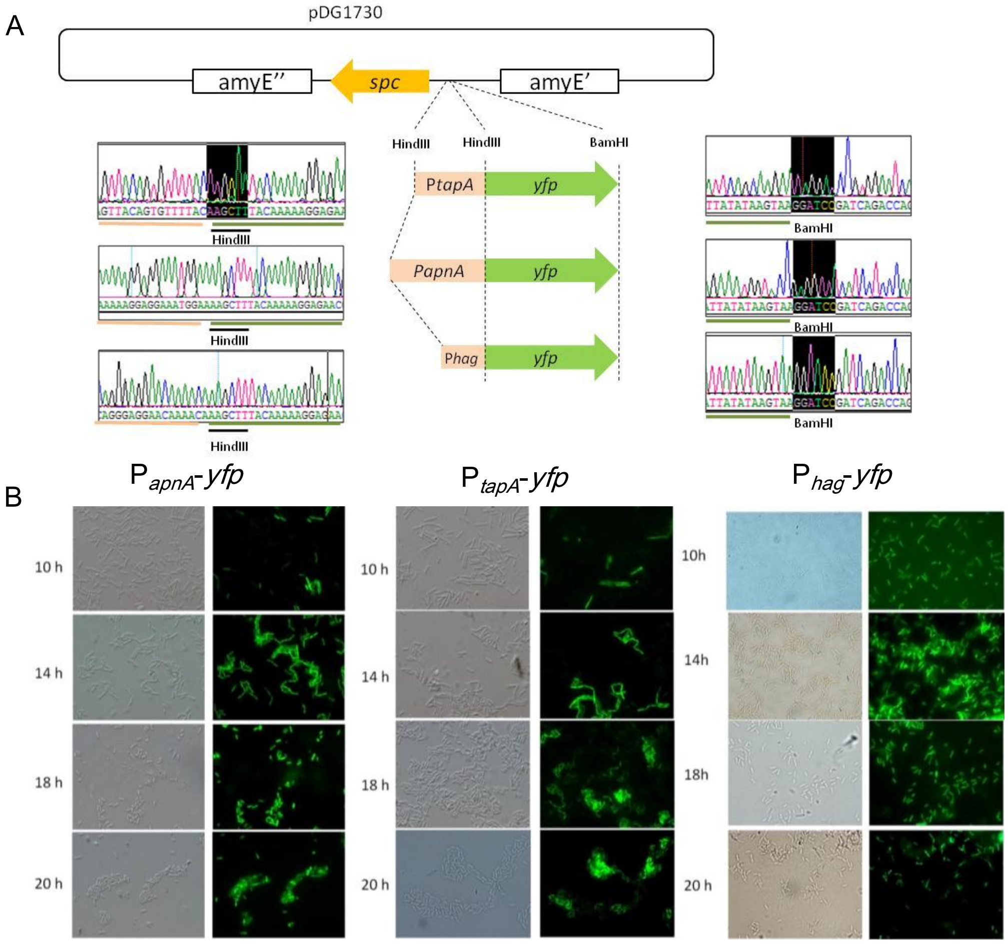

Supplementary Fig. 11 Verification of the BSn5 derived *yfp* reporter strains. (A) Identification the construction of the vectors of P_{tapA}-*yfp*-pDG1730, P_{apnA}-*yfp*-pDG1730, and P_{hag}-*yfp*-pDG1730 by sequencing. (B) The expression of gene *yfp* in BSn5 derived reporter strains PapnA-*yfp*, PtapA-*yfp*, and Phag-*yfp* under fluorescence microscope.

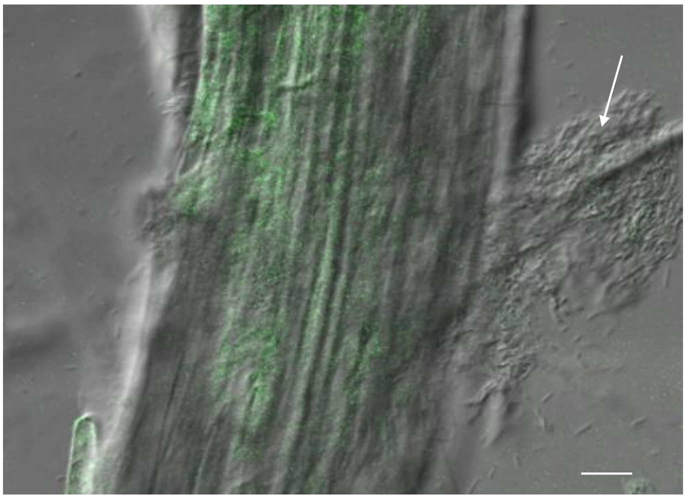

Supplementary Fig. 12 Observation of the wild type strain BSn5 without YFP labeling after 36 h inoculating in *A.thaliana* under fluorescence microscope. The arrow indicates the bacteria. Scare bar: 20 μm.

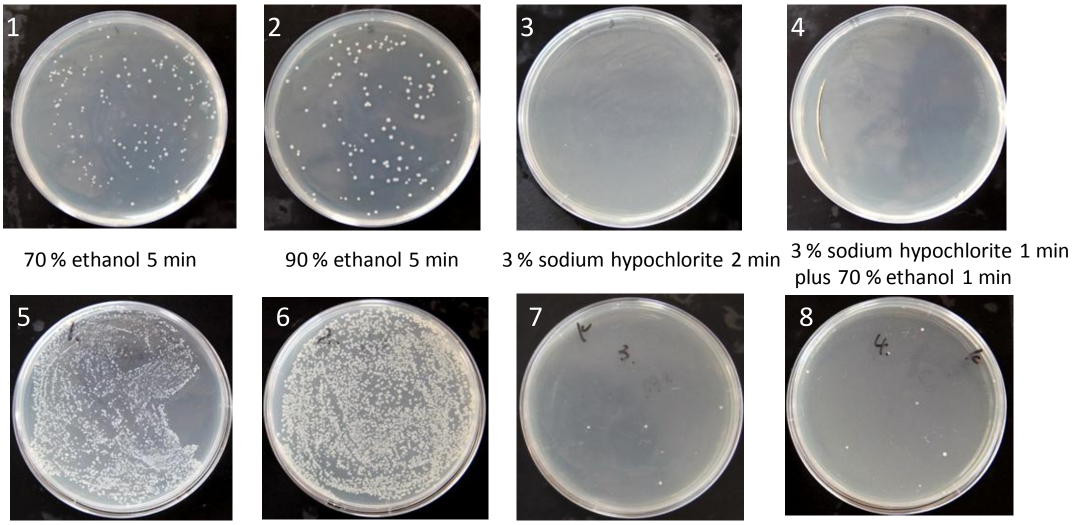

Supplementary Fig. 13 The comparison of the surface disinfection treatments to *Arabidopsis* seedlings after incubating with *B. subtilis* and culturing for 7 days. After treatments by mixing in 70 % ethanol for 5 min, mixing in 90 % ethanol for 5 min, mixing in 3 % sodium hypochlorite for 2 min, shake in 3 % sodium hypochlorite for 1 min plus 70 % ethanol for 1min, respectively, and 1 time washing with sterile saline, the 200 μL supernatant after vortex for 30 sec with another 1mL sterile saline were respectively used for spreading plates 1-4 and the seedlings after washing were collected for grinding. The 200 μL homogenate in sterile saline were respectively used for spreading the plate 5-8. The treatment of mixing in 3 % sodium hypochlorite for 2 min was chosen as the surface disinfection procedure.

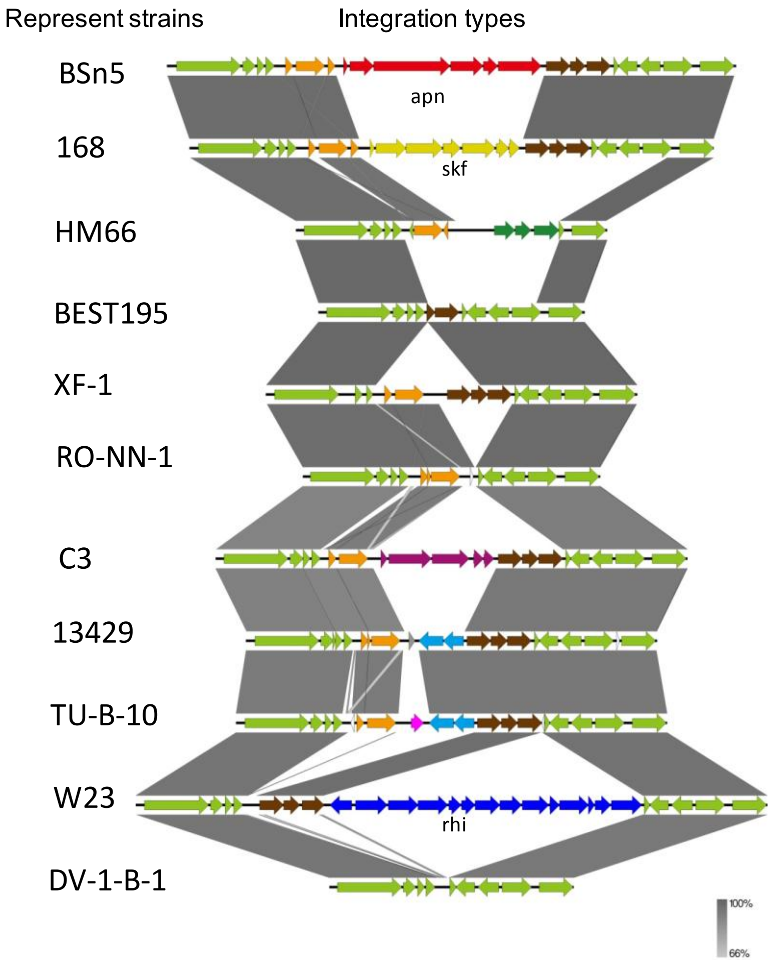

Supplementary Fig. 14 The comparison of apn-locus in different strains of *Bacillus subtilis* species. The apn refers to subtilomycin gene cluster in BSn5. The skf refers to sporulation killing factors gene cluster in 168. The rhi refers to rhizocticins gene cluster in W23.

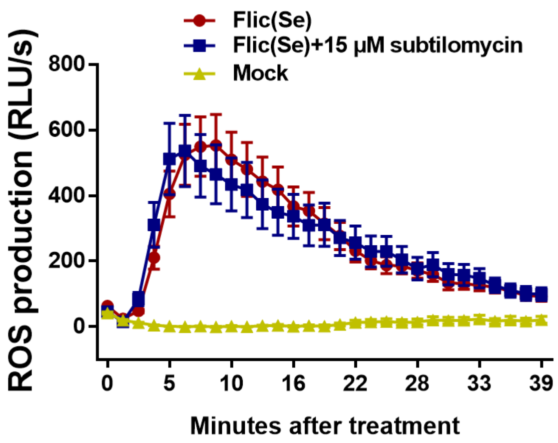

Supplementary Fig. 15 The ROS induced by the 30 μM FliC (Se) from *Salmonella enteritidis* and FliC (Se) pre-mixed with 15 μM subtilomycin through luminescence assay. Error bars are standard errors, n=8.

Supplementary Table 1 Identification the plots of the Apn5 protein from 2D SDS-PAGE by Mass spectrum.

No.	Protein description	NCBI entry	Score	Expect	Queries matched	Source species
1	flagellin	gi|14278890	46	0.15	7	*Bacillus subtilis*
2	hypothetical protein BSU33620	gi|16080415	38	1.1	6	*Bacillus subtilis*
3	flagellin	gi|14278890	53	0.131	6	*Bacillus subtilis*
4	flagellin	gi|14278890	62	0.0044	8	*Bacillus subtilis*
5	flagellin	gi|14278890	63	0.0031	9	*Bacillus subtilis*
6	flagellin	gi|14278890	55	0.019	10	*Bacillus subtilis*
7	hypothetical protein BSU33670	gi|16080420	43	0.33	5	*Bacillus subtilis*
8	RNA polymerase sigma-54 factor (sigma-L)	gi|16080473	48	0.11	11	*Bacillus subtilis*
9	hypothetical protein BSU23670	gi|16079424	35	2.3	7	*Bacillus subtilis*
10	Protopectinase-R, endo-pectin transeliminase, pectin lyase, PPase-R	gi|545812	32	1.1	3	*Bacillus subtilis*
11	Hypothetical 12.2 kDa protein	gi|1673391	48	0.097	7	*Bacillus subtilis*

Supplementary Table 2 The mining of subtilomycin producer strains with corresponding isolation source from NCBI database (Updated to June 2019)

No.	Strains	Species	Isolation origin	Identities to apn cluster (coverage)	location	GenBank accession numbers
1	BSn5	*B.subtilis*	*Amorphophallus konjac*	100 %（100 %）	2428598-2436872	CP002468.1
2	OH131.1	*B.subtilis*	The anthers of wheat	99 %（100 %）	202870-211144	gb|CP007409.1|
3	CGMCC 63528	*B.subtilis*	Soil	99 %（100 %）	169790-161516*	gb|JWHO01000009.1|
4	UMX-103	*B.subtilis*	Soil	99 %（100 %）	37303-45577	dbj|BDCV01000005.1|
5	HJ0-6	*B.subtilis*	Fermented soybean paste	99 %（100 %）	213105-221379	CP016894.1
6	SRCM101280	*B.subtilis*	Doenjang	100 %（100 %）	37709-45983	gb|LYUI01000022.1|
7	BGSC 3A28	*B.subtilis*	Agave microbiome	100 %（100 %）	386193-377919*	emb|FODG01000004.1|
8	MMA7	*B.subtilis*	The marine sponge *Haliclona simulans*	99 %（100 %）	832-9106	JX912247.1
9	FJAT-10019	*B.subtilis*	Soil	99 %（100 %）	3086860-3095134	CP017070.1
10	FJAT-14266	*B.subtilis*	Soil	99 %（100 %）	4057605-4049331*	NZ_CP017072.1
11	FJAT-14262a	*B.subtilis*	Soil	99 %（100 %）	37514-45788	gb|LGRW01000004.1|
12	DJHJ8	*B.atrophaeus*	Soybean paste	94 % (97 %)	39688-48565	dbj|BDDH01000005.1|
13	QB61	*B.subtilis*	Soil	100 %（100 %）	121206-132244	CP029461.2
14	MG27	*B.subtilis*	Soil	100 %（100 %）	34760-45809	QJJA01000004.1
15	SW83	*B.subtilis*	plant *Turbinaria ornata*	98 %（100 %）	173177-184200	AXC51530.1
16	IITK SM1	*B.subtilis*	food waste compost	99 %（100 %）	150061-161110	CP031675.1
17	SSJ-1	*B.subtilis*	Fermented soybean paste	99 %（100 %）	1766593-1777617	CP032860.1

* indicates that the sequences are reverse direction to the apn cluster

Supplementary Table 3. Investigation of subtilomycin production on *Bacillus subtilis* isolates

No.	Strains	Species	Origin	*apn* detetion by PCR	Subtilomycin production by LC-MS	Sample name
1	3-1	*B.subtilis*	Tianyang, Guangxi, Soil	No	No	c-2
2	3-3	*B.subtilis*	Tianyang, Guangxi, Soil	Yes	Yes	c-2
3	3-6	*B.subtilis*	Tianyang, Guangxi, Soil	No	No	c-2
4	3-20	*B.subtilis*	Tianyang, Guangxi, Soil	Yes	Yes	c-3
5	3-22	*B.subtilis*	Tianyang, Guangxi, Soil	Yes	Yes	c-3
6	3-25	*B.subtilis*	Tianyang, Guangxi, Soil	No	No	c-6
7	3-31	*B.subtilis*	Xining, Qinghai, Soil	No	No	c-16
8	3-36	*B.subtilis*	Xining, Qinghai, Soil	No	No	c-17
9	3-37	*B.mojavensis*	Xining, Qinghai, Soil	No	No	c-17
10	3-42	*B.subtilis*	Yulin, Guangxi, Soil	No	No	c-5
11	3-47	*B.subtilis*	Wuzhou, Guangxi, Soil	No	No	c-1
12	3-58	*B.subtilis*	Gangcha, Qinghai, Soil	No	No	c-12
13	3-59	*B.subtilis*	Gangcha, Qinghai, Soil	No	No	c-12
14	3-69	*B.subtilis*	Gangcha, Qinghai, Soil	No	No	c-10
15	4-4	*B.amyloliquefaciens*	Fengan, Guizhou, Soil	No	No	c-45
16	4-6	*B.mojavensis*	Fengan, Guizhou, Soil	No	No	c-42
17	4-9	*B.mojavensis*	Ledu, Qinghai, Soil	No	No	c-41
18	4-10	*B.mojavensis*	Ledu, Qinghai, Soil	No	No	c-41
19	4-11	*B.mojavensis*	Ledu, Qinghai, Soil	No	No	c-40
20	4-12	*B.subtilis*	Ledu, Qinghai, Soil	No	No	c-40
21	4-13	*B.mojavensis*	Ledu, Qinghai, Soil	No	No	c-39
22	4-14	*B.subtilis*	Ledu, Qinghai, Soil	No	No	c-39
23	4-15	*B.mojavensis*	Ledu, Qinghai, Soil	No	No	c-39
24	4-16	*B.mojavensis*	Ledu, Qinghai, Soil	No	No	c-38
25	4-17	*B.subtilis*	Ledu, Qinghai, Soil	No	No	c-38
26	4-18	*B.mojavensis*	Ledu, Qinghai, Soil	No	No	c-38
27	4-19	*B.atrophaeus*	Ledu, Qinghai, Soil	No	No	c-38
28	4-21	*B.atrophaeus*	Gonghe, Qinghai, Soil	No	No	c-29
29	4-22	*B.atrophaeus*	Gonghe, Qinghai, Soil	No	No	c-30
30	4-24	*B.atrophaeus*	Gonghe, Qinghai, Soil	No	No	c-28
31	4-25	*B.mojavensis*	Gonghe, Qinghai, Soil	No	No	c-32
32	4-27	*B.mojavensis*	Gonghe, Qinghai, Soil	No	No	c-32
33	4-28	*B.mojavensis*	Gonghe, Qinghai, Soil	No	No	c-32
34	4-30	*B.mojavensis*	Gonghe, Qinghai, Soil	No	No	c-33
35	4-31	*B.mojavensis*	Gonghe, Qinghai, Soil	No	No	c-36
36	4-32	*B.mojavensis*	Gonghe, Qinghai, Soil	No	No	c-36
37	4-34	*B.mojavensis*	Gonghe, Qinghai, Soil	No	No	c-36
38	4-36	*B.mojavensis*	Gonghe, Qinghai, Soil	No	No	c-35
39	4-37	*B.subtilis*	Gonghe, Qinghai, Soil	No	No	c-35
40	4-38	*B.mojavensis*	Gonghe, Qinghai, Soil	No	No	c-35

41	4-39	*B.atrophaeus*	Gonghe, Qinghai, Soil	No	No	c-34
42	4-40	*B.mojavensis*	Gonghe, Qinghai, Soil	No	No	c-34
43	4-41	*B.subtilis*	Gonghe, Qinghai, Soil	No	No	c-34
44	7-2	*B.subtilis*	HZAU, Wuhan, Plant[c], Mulberry	No	No	p-1
45	7-7	*B.subtilis*	HZAU[a], Wuhan, Plant, Mulberry	Yes	Yes	p-1
46	10-1	*B.subtilis*	HZAU, Wuhan, Plant *Cotula anthemoides*	Yes	Yes	p-10
47	10-15	*B.mojavensis*	HZAU, Wuhan, Plant *Rhododendron*	No	No	P-15
48	10-16	*B.subtilis*	HZAU, Wuhan, Plant *Rhododendron*	Yes	Yes	P-15
49	11-1	*B.subtilis*	HZAU, Wuhan, Plant Speedwell	Yes	Yes	P-17
50	11-2	*B.subtilis*	HZAU, Wuhan, Plant Speedwell	Yes	Yes	P-17
51	11-4	*B.mojavensis*	HZAU, Wuhan, Plant Chinese violet	Yes	Yes	P-18
52	11-5	*B.subtilis*	HZAU, Wuhan, Plant Chinese violet	Yes	Yes	P-18
53	11-6	*B.mojavensis*	HZAU, Wuhan, Plant Chinese violet	Yes	Yes	P-18
54	11-8	*B.subtilis*	HZAU, Wuhan, Plant undetermined	Yes	Yes	P-19
55	12-2	*B.subtilis*	HZAU, Wuhan, Plant *Euphorbia helioscopia*	Yes	Yes	p-20
56	12-3	*B.subtilis*	HZAU, Wuhan, Plant *Euphorbia helioscopia*	Yes	Yes	p-20
57	12-4	*B.subtilis*	HZAU, Wuhan, Plant *Euphorbia helioscopia*	Yes	Yes	p-20
58	13-1	*B.subtilis*	HZAU, Wuhan, Plant *Conyza canadensis*	Yes	Yes	p-21
59	13-3	*B.subtilis*	HZAU, Wuhan, Plant *Euphorbia helioscopia*	Yes	Yes	p-22
60	13-4	*B.subtilis*	HZAU, Wuhan, Plant undetermined	Yes	Yes	p-23
61	P01	*B.amyloliquefaciens*	HUST[b], Wuhan, Plant undetermined	No	No	p-30
62	P02	*B.subtilis*	HUST, Wuhan, Plant undetermined	Yes	Yes	p-30
63	P03	*B.subtilis*	HUST, Wuhan, Plant undetermined	Yes	Yes	p-31
64	P04	*B.subtilis*	HUST, Wuhan, Plant	Yes	Yes	p-31

			undetermined			
65	P05	*B.subtilis*	HUST, Wuhan, Plant undetermined	No	No	p-31
66	P08	*B.amyloliquefaciens*	HUST, Wuhan, Plant undetermined	No	No	p-32
67	P09	*B.subtilis*	Turtle mountain, Wuhan, Plant *Cyclosorus acuminatus*	Yes	Yes	p-40
68	P10	*B.subtilis*	Turtle mountain, Wuhan, Plant *Ficus tikoua*	Yes	Yes	p-41
69	P11	*B.subtilis*	Turtle mountain, Wuhan, Plant *Rhododendron*	Yes	Yes	p-42
70	P12	*B.amyloliquefaciens*	Turtle mountain, Wuhan, Plant undetermined	No	No	p-43
71	P13	*B.subtilis*	HUST, Wuhan, Plant undetermined	Yes	Yes	p-46
72	P14	*B.subtilis*	HUST, Wuhan, Plant undetermined	Yes	Yes	p-46
73	P15	*B.subtilis*	HUST, Wuhan, Plant undetermined	Yes	Yes	p-46
74	P16	*B.subtilis*	HUST, Wuhan, Plant undetermined	Yes	Yes	p-47
75	P17	*B.subtilis*	HUST, Wuhan, Plant undetermined	Yes	Yes	p-47
76	P18	*B.subtilis*	HUST, Wuhan, Plant *Pronephrium simplex*	Yes	Yes	p-48
77	P19	*B.subtilis*	HUST, Wuhan, Plant *Pronephrium simplex*	Yes	Yes	p-48
78	P20	*B.subtilis*	HUST, Wuhan, Plant *Pronephrium simplex*	Yes	Yes	p-48
79	P21	*B.subtilis*	HUST, Wuhan, Plant *Pronephrium simplex*	Yes	Yes	p-48
80	P22	*B.subtilis*	HUST, Wuhan, Plant *Pronephrium simplex*	Yes	Yes	p-48
81	P23	*B.subtilis*	HUST, Wuhan, Plant *Brassica oleracea*	Yes	Yes	p-49
82	P24	*B.subtilis*	HUST, Wuhan, Plant *Lycoris*	Yes	Yes	p-50
83	P25	*B.subtilis*	HUST, Wuhan, Plant undetermined	Yes	Yes	p-51
84	P26	*B.subtilis*	HUST, Wuhan, Plant undetermined	Yes	Yes	p-51
85	P27	*B.subtilis*	HUST, Wuhan, Plant undetermined	Yes	Yes	p-51

86	P28	*B.amyloliquefaciens*	HUST, Wuhan, Plant undetermined	No	No	p-51
87	P29	*B.subtilis*	HUST, Wuhan, Plant undetermined	Yes	Yes	p-52
88	P30	*B.subtilis*	HUST, Wuhan, Plant undetermined	Yes	Yes	p-52
89	P31	*B.subtilis*	HUST, Wuhan, Plant undetermined	Yes	Yes	p-52
90	P32	*B.subtilis*	HUST, Wuhan, Plant undetermined	Yes	Yes	p-52
91	XJY13	*B.subtilis*	Soil	No	No	c-55
92	XJY16	*B.subtilis*	Soil	No	No	c-56
93	XJY5	*B.mojavensis*	Soil	No	No	c-57

[a] HZAU refers to Huazhong Agricultural University.

[b] HUST refers to Huazhong University of Science and Technology.

[c] The recognition of plants are based on uploading the photos on PPBC (http://www.plantphoto.cn) or knowledge of sampling people.

Supplementary Table 4. The mining of subtilomycin analogues producer strains with corresponding isolation source from NCBI database (Updated to June 2019)

No.	Strains	Species	Isolation origin	Identities to apnA (coverage)	Identities to ApnB (coverage)	GenBank accession numbers
1	BAG1X1-1	*B. cereus*	NG	51-56%（94%）	36-37% (98%)	AHCQ01000073.1
2	BAG1X2-1	*B. cereus*	NG	51-56%（94%）	36-37% (98%)	AHCT01000095.1
3	BAG1X2-2	*B. cereus*	NG	51-56%（94%）	36-37% (98%)	AHCU01000046.1
4	BAG1X2-3	*B. cereus*	NG	51-56%（94%）	36-37% (98%)	AHCV01000030.1
5	BAG2O-1	*B. cereus*	NG	51-56%（94%）	36-37% (98%)	AHCW01000050.1
6	NA205-3	*B. thuringiensis*	dust from maize silo	51-56%（94%）	36-37% (98%)	AYXQ01000049.1
7	T01001	*B. thuringiensis*	insect	51-56%（94%）	36-37% (98%)	KP133062.1
8	IBL200	*B. thuringiensis*	insect	51-56%（94%）	36-37% (98%)	ACNK01000131.1
9	10792	*B. thuringiensis*	insect	51-56%（94%）	36-37% (98%)	CP020755.1
10	T01-328	*B. thuringiensis*	soil	51-56%（94%）	36-37% (98%)	ARXZ02000035.1
11	IS5056	*B. thuringiensis*	insect Trichoplusia ni	51-56%（94%）	36-37% (98%)	CP004135.1
12	BGSC 4A4	*B. thuringiensis*	insect Ephestia elutella	51-56%（94%）	36-37% (98%)	NFCE01000078.1
13	BGSC 4N1	*B. thuringiensis*	insect Galleria mellonella	51-56%（94%）	36-37% (98%)	NFEB01000063.1 &NFEB01000127.1
14	BGSC 4R1	*B. thuringiensis*	NG	51-56%（94%）	36-37% (98%)	MOOT01000054.1
15	AFS095989	*B. thuringiensis*	Soybean core	51-56%（94%）	36-37% (98%)	NVLS01000053.1
16	AFS088784	*B. cereus*	Corn	51-56%（94%）	36-37% (98%)	NVNN01000071.1
17	AFS093925	*B. cereus*	Corn	51-56%（94%）	36-37% (98%)	NVML01000001.1
18	AFS093334	*B. thuringiensis*	Soybean	51-56%（94%）	36-37% (98%)	NVNS01000090.1 &NVNS01000039.1
19	AFS089527	*B. cereus*	Soybean	51-56%（94%）	36-37% (98%)	NVNK01000020.1
20	AFS005998	*B. cereus*	Soybean	51-56%（94%）	36-37% (98%)	NTYA01000034.1
21	AFS009411	*B. cereus*	Soybean	51-56%（94%）	36-37% (98%)	NTWP01000068.1 &NTWP01000083.1
22	AFS017211	*B. cereus*	Soybean	51-56%（94%）	36-37% (98%)	NTUA01000043.1
23	AFS020602	*B. thuringiensis*	soil	51-56%（94%）	36-37% (98%)	NTSL01000146.1 &NTSL01000350.1 & NTSL01000171.1
24	AFS029513	*B. cereus*	Soybean	51-56%（94%）	36-37% (98%)	NTSQ01000055.1
25	AFS087824	*B. cereus*	Soybean	51-56%（94%）	36-37% (98%)	NUVA01000069.1 &NUVA01000056.1
26	AFS081512	*B. cereus*	Soybean	51-56%（94%）	36-37% (98%)	NUXB01000072.1
27	AFS073631	*B. cereus*	Soybean	51-56%（94%）	36-37% (98%)	NUZQ01000008.1
28	AFS071044	*B. cereus*	Soybean	51-56%（94%）	36-37% (98%)	NVAK01000025.1 & NVAK01000093.1
29	AFS070860	*B. cereus*	Soybean	51-56%（94%）	36-37% (98%)	NVAQ01000050.1

30	AFS068756	*B. cereus*	Soybean	51-56%（94%）	36-37% (98%)	NVBA01000086.1 & NVBA01000097.1
31	AFS068289	*B. cereus*	Soybean	51-56%（94%）	36-37% (98%)	NVBE01000006.1 &NVBE01000090.1
32	AFS065816	*B. cereus*	Weed leaf	51-56%（94%）	36-37% (98%)	NVCG01000055.1
33	AFS065666	*B. thuringiensis*	soil	51-56%（94%）	36-37% (98%)	NVCK01000032.1
34	AFS057244	*B. thuringiensis*	soil	51-56%（94%）	36-37% (98%)	NUFW01000077.1
35	AFS056399	*B. cereus*	Soybean core	51-56%（94%）	36-37% (98%)	NUGF01000099.1 &NUGF01000099.1
36	AFS053259	*B. cereus*	Soybean core	51-56%（94%）	36-37% (98%)	NUHM01000026.1
37	AFS036437	*B. thuringiensis*	mashroom	51-56%（94%）	36-37% (98%)	NUNG01000052.1 &NUNG01000081.1 &NUNG01000123.1
38	AFS029810	*B. cereus*	Soybean root	51-56%（94%）	36-37% (98%)	NUPV01000055.1
39	BAM	*B. thuringiensis*	soil	51-56%（94%）	36-37% (98%)	QUQW01000020.1
40	MRIK1	*B. cereus*	soil	51-56%（94%）	36-37% (98%)	PKRK01000025.1
41	AFS006766	*B. cereus*	Weed leaf	51-56%（94%）	36-37% (98%)	NTXS01000045.1 &NTXS01000076.1
42	AFS067996	*B. cereus*	Corn core	51-56%（94%）	36-37% (98%)	NVBF01000055.1 &NVBF01000091.1 &NVBF01000122.1
43	AFS058833	*B. cereus*	Corn core	51-56%（94%）	36-37% (98%)	NVEM01000049.1 & NVEM01000056.1
44	AFS057725	*B. cereus*	soil	51-56%（94%）	36-37% (98%)	NUQH01000009.1
45	AFS029792	*B. cereus*	soil	51-56%（94%）	36-37% (98%)	NUUR01000128.1 &NUUR01000186.1
46	DB-2	*Bacillus*	NG	51-56%（94%）	37% (98%)	QNRN01000010.1
47	DB-1	*B.mycoides*	NG	51-56%（94%）	37% (98%)	QTTY01000014.1
48	AFS085794	*B. cereus*	Corn leaf	51-56%（94%）	36-37% (98%)	NUVT01000005.1
49	AFS039342	*B. cereus*	Corn leaf	51-56%（94%）	36-37% (98%)	NUMR01000107.1
50	AFS020602	*B. thuringiensis*	soil	51-56%（94%）	36-37% (98%)	NTSL01000146.1 &NTSL01000350.1 & NTSL01000171.1
51	AFS065666	*B. thuringiensis*	soil	51-56%（94%）	36-37% (98%)	NVCK01000032.1
52	AFS057244	*B. thuringiensis*	soil	51-56%（94%）	36-37% (98%)	NUFW01000077.1
53	AFS008265	*B. cereus*	Corn	51-56%（94%）	36-37% (98%)	NTXC01000432.1 &NTXC01000099.1 & NTXC01000581.1& NTXC01000426.1& NTXC01000512.1
54	AFS053130	*B. cereus*	soil	51-56%（94%）	36-37% (98%)	NUHO01000077.1

55	OSY-DF	*Paenibacillus polymyxa*	fermented vegetable (Kimchee) from a local store	47%（96%）	29% (99%)	AIPP01000129.1
56	ND25	*Paenibacillus polymyxa*	cow rumen	47%（96%）	29% (99%)	LZEL01000038.1
57	A18	*Paenibacillus polymyxa*	insect termite gut	47%（96%）	29% (99%)	JWJJ01000002.1
58	Mc5Re-14	*Paenibacillus*	Matricaria chamomilla endorhiza	47%（96%）	29% (99%)	CVPD01000001.1
59	ICGEB2008	*Paenibacillus*	gut of Helicoverpa armigera	47%（96%）	29% (99%)	AMQU01000007.1
60	ATCC 842	*Paenibacillus polymyxa*	NG	47%（96%）	29% (99%)	AFOX01000014.1
61	KCCM 40454	*Paenibacillus polymyxa*	soil	47%（96%）	29% (99%)	POVT01000017.1
62	KP 10	*Paenibacillus polymyxa*	milk curd	47%（96%）	29% (99%)	QAOE01000012.1
63	NCTC10343	*Paenibacillus polymyxa*	NG	47%（96%）	29% (99%)	UGSC01000001.1
64	KACC 10925	*Paenibacillus jamilae*	NG	47%（96%）	29% (99%)	QVPU01000001.1
65	CFSAN034341	*Paenibacillus polymyxa*	agricultural soil	47%（96%）	29% (99%)	LYMX01000188.1
66	DSM 365	*Paenibacillus polymyxa*	garden soil	47%（96%）	29% (99%)	JMIQ01000006.1
67	WLY78 S6	*Paenibacillus polymyxa*	soil	47%（96%）	29% (99%)	ALJV01000177.1 & ALJV01000176.1
68	DMB5	*Paenibacillus*	polluted soil	52%(94%)	29% (99%)	LRAC01000120.1

Supplementary Table 5. Plasmids used in this study

Plasmid name	Vector	Purpose	Reference
pB1206	pMD18T	Gene *hag* knock out by homologous recombination of mutation *hag::spc* in *Bacillus subtilis* BSn5	This study
pMD18T-hag	pMD18T(simple)	Cloning of gene *hag* into vector pMD18T-simple	This study
pB1208	pDG780	Gene *ymdB* knock out by homologous recombination of mutation *ymdB::kan* in *Bacillus subtilis* BSn5	This study
pB1311	pDG1730	Construction of YFP reporter strain Phag-yfp by integration into the *amyE* locus in *Bacillus subtilis* BSn5	This study
pB1312	pDG1730	Construction of YFP reporter strain PapnA-yfp by integration into the *amyE* locus in *Bacillus subtilis* BSn5	This study
pB1313	pDG1730	Construction of YFP reporter strain PtapA-yfp by integration into the *amyE* locus in *Bacillus subtilis* BSn5	This study
pB1101	pET28a	Expression of Hag in *Escherichia coli* for purification	This study
pB1107	pET28a	Expression of ΔHag (flg22) in *Escherichia coli* for purification	This study
pB1108	pET28a	Expression of Hag (ex) in *Escherichia coli* for purification	This study

Supplementary Table 6. Primers used in this study

Primer name	Primer 5'-3'*	Purpose
gyrAF	CAGTCAGGAAATGCGTACGTCCTT	Identification of wild isolated strain
gyrAR	CAAGGTAATGCTCCAGGCATTGCT	Identification of wild isolated strain
apnF	AGTTTGGTTGGATCAAAG	Investigation the presence of subtilomycin gene on *B. subtilis* isolates
apnR	TTCCATTTCCTCCTTTT	Investigation the presence of subtilomycin gene on *B. subtilis* isolates
hagF	GCTGGATCCATCGCGGAAAATAAGCGAAGC	Clone gene *hag* from BSn5 for gene disruption
hagR	CAGTGTCGACCGCATAAAAATCAAGCCACC	Clone gene *hag* from BSn5 for gene disru ption
SpcF	AGTATCGATGCGGTGCTACAGAGTTCTTG	Amplification of *spectinomycin* resistance gene from plasmid pIC333
SpcR	GGGTCTAGAGTAAACGCTGAATATCGTGTT	Amplification of *spectinomycin* resistance gene from plasmid pIC333
YmdBUpF	CGATGGATCCATTCGTGAGATGGGTGAG	*ymdB* gene disruption
YmdBUpR	ATCTGAATTCGACTGGATTAGGCTGTGA	*ymdB* gene disruption
YmdBDownF	GACGTCGACCGTCGGCTGTAGTAGGAA	*ymdB* gene disruption
YmdBDownF	GCAAGGTACCCTGCCACTGAATTTGGAC	*ymdB* gene disruption
Hag Exp-F	GCCGGATCCAGAATTAACCACAATATCGCAGC	Expression of His-tag Hag protein
Hag Exp-R	GCCCTCGAGACGTAATAATTGAAGTACG	Expression of His-tag Hag protein
reverse-del-flg 22-1	AAAGAATTCGAAAAAATGAGAGG	Construction truncated His-tag protein Hag (del-flg22) for expression and purification
reverse-del-flg 22-2	CCCGAATTCCATGTTTTTTTGTGATGCACCG	Construction truncated His-tag protein Hag (del-flg22) for expression and purification
reverse-del-flg 22-3	GAAAAAATGAGAGGACAAATC	Construction reconstructed His-tag protein Hag (ex) for expression and purification
reverse-del-flg 22-4	CATGTTTTTTTGTGATGCACCG	Construction reconstructed His-tag protein Hag (ex) for expression and purification
flg22(ps)-hag-1	CGGTGCATCACAAAAAAACATGACTCGTCTGTCCTCCGG	Construction reconstructed His-tag protein Hag (ex) for expression and purification
flg22(ps)-hag-2	GATTTGTCCTCTCATTTTTTCAGCGATTTGCAGGCCGGC	Construction reconstructed His-tag protein Hag (ex) for expression and purification
UIBQ10-f	GGCCTTgTATAATCCCTGATGAATAAG	qRT-PCR analysis
UIBQ10-f	AAAGAGATAACAGGAACGGAAACATAG	qRT-PCR analysis
FRK1-f	TGCAGCGCAAGGACTAGAG	qRT-PCR analysis
FRK1-r	ATCTTCGCTTGGAGCTTCTC	qRT-PCR analysis
yfp-F	CCCAAGCTTTACAAAAAGGAGAACGCATAATGTCAAAAGGAGAAGAATTATTTACAG	Sub-clone of gene *yfp* from the vector pSW4-yfp(opt) into pDG1730
yfp-R	CGCGGATCCTTACTTATATAATTCATCCATTCCGTG	Sub-clone of gene *yfp* from the vector pSW4-yfp (opt) into pDG1730
protapA-F	TGGCGAATTCTCAGAGTTAAATGGTATTGCTTCACT	Construction PtapA-yfp-1730
protapA-R	GCCTAAGCTTGTAAAACACTGTAACTTGATATGACAA	Construction PtapA-yfp-1730
proapnA-F	TTTAAGCTTGCGAGCTGAAGTACAGTACG	Construction PapnA-yfp-1730
proapnA-R	CTTAAGCTTTTCCATTTCCTCCTTTT	Construction PapnA-yfp-1730
prohag-F	TTTAAGCTTTGCTAGTTTTATCGCGG	Construction Phag-yfp-1730
prohag-R	CTTAAGCTTTGTTTTGTTCCTCCCTG	Construction Phag-yfp-1730

* Underline refers to restriction enzyme cutting site.
