# Supplementary material for: Endophyte Bacillus subtilis evade plant defense by producing lantibiotic subtilomycin to mask self-produced flagellin
Source: Commun Biol. 2019 Oct 10;2:368. doi: 10.1038/s42003-019-0614-0 (PMC6787100; doi:10.1038/s42003-019-0614-0)
Supplement: Supplementary file 2 — Description of Additional Supplementary Files [file 42003_2019_614_MOESM2_ESM.pdf]

Descriptions of Additional Supplementary Files
File Name: Supplementary Data 1
Description: Source data file

Fig. 1e Source data for the size exclusion chromatography assay of Apn5. Inhibition holo r(mm) indicates the relative activity of subtilomycin. Quantification for CBB stain band by software Quantity One (Bio-Rad).

Fig.1g Source data for MST analysis of the interaction of His-tagged Hag and purified subtilomycin.

Fig. 2b Source data for detection of ROS production in *A. thaliana* Col-0 and fls2-1 treated by Hag, Hag (flg22), and Hag mixed with 15 μM subtilomycin through luminescence assay.

Fig. 2c Source data for detection of ROS production in *A. thaliana Col-0* treated by Hag, Apn5 protein and ddH2O (Mock) through luminescence assay.

Fig 2d Source data for detection of Hag induced ROS production in wild *A. thaliana* Col-0 leaf strips. The gradient concentrations of subtilomycin 7.5 μM, 15 μM, and 30 μM were applied to pre-incubate with Hag, respectively.

Fig. 2e Source data for subtilomycin attenuate Hag induced stomatal closure response. Stomatal response induced by different elicitors, Hag, Hag with subtilomycin, and flg22 (Ps) for 1 h in Col-0.

Fig. 2g Source data for inhibition of Hag-induced *frk1::LUC* expression by subtilomycin in wild type *A. thaliana* Col-0.

Fig. 3b Source data for detection of Hag (ex) induced *frk1-LUC* activity at the present or absent of subtilomycin, respectively.

Fig. 3c Source data for detection of flg22(Ps)and flg22(Bs) induced *frk1-LUC* activity at the present or absent of subtilomycin, respectively.

Fig. 3d-3f Source data for detection the ROS production induced by Hag, Hag (ex), and flg22 (Bs) at the presence or absence of subtilomycin, respectively.

Fig. 3g Source data for detection the ROS production induced by flg22 (Ps) at the presence or absence of subtilomycin, respectively.

Fig. 3h-k Source data for MST assays on the interaction of subtilomycin with the peptides ΔHag (del-flg22), Hag (ex), flg22 (Bs) and flg22 (Ps), respectively.

Fig. 4e and 4f Source data for colonization assay *B. subtilis* BSn5, subtilomycin mutant apnB, and biofilm mutant ymdB in the rhizoplane, and endosphere of *A. thaliana* Col-0 and fls2-1 seedlings, respectively.

Fig. 5a and 5b Source data for colonization assay *B. subtilis* BSn5, subtilomycin mutant apnB, and biofilm mutant ymdB in the rhizosphere soil from roots and root, respectively.

Supplementary Fig. 8 Source data for the ROS induced by the 2 μM flg22 peptide from *Pseudomonas syringae,* flg22 (Ps), and the gradient concentrations (7.5~60 μM) of flg22 peptide from *B. subtilis*, flg22 (Bs).

Supplementary Fig. 9a Source data for inhibition of Hag-induced *frk1* gene expression by subtilomycin in wild type *A. thaliana* Col-0 and mutant fls2-1 by qRT-PCR.

Supplementary Fig. 9b Source data for inhibition of Hag-induced *frk1::LUC* activity by subtilomycin in wild type A. thaliana Col-0 leaf protoplasts using dual reporter system.

Supplementary Fig. 15 Source data for the ROS induced by the 30 μM FliC (Se) from *Salmonella enteritidis* and FliC (Se) pre-mixed with 15 μM subtilomycin through luminescence assay.
