# Supplementary material for: Endophyte Bacillus subtilis evade plant defense by producing lantibiotic subtilomycin to mask self-produced flagellin
Source: Commun Biol. 2019 Oct 10;2:368. doi: 10.1038/s42003-019-0614-0 (PMC6787100; doi:10.1038/s42003-019-0614-0)
Supplement: Supplementary file 4 — Reporting Summary [file 42003_2019_614_MOESM4_ESM.pdf]

nature research

Corresponding author(s): Sun, Ming

Last updated by author(s): Aug 16, 2019

Reporting Summary

Nature Research wishes to improve the reproducibility of the work that we publish. This form provides structure for consistency and transparency in reporting. For further information on Nature Research policies, seeAuthors & Referees and theEditorial Policy Checklist .

Statistics

For all statistical analyses, confirm that the following items are present in the figure legend, table legend, main text, or Methods section.

n/a	Confirmed	
☐	☒	The exact sample size (*n*) for each experimental group/condition, given as a discrete number and unit of measurement
☐	☒	A statement on whether measurements were taken from distinct samples or whether the same sample was measured repeatedly
☐	☒	The statistical test(s) used AND whether they are one- or two-sided<br>*Only common tests should be described solely by name; describe more complex techniques in the Methods section.*
☒	☐	A description of all covariates tested
☐	☒	A description of any assumptions or corrections, such as tests of normality and adjustment for multiple comparisons
☐	☒	A full description of the statistical parameters including central tendency (e.g. means) or other basic estimates (e.g. regression coefficient) AND variation (e.g. standard deviation) or associated estimates of uncertainty (e.g. confidence intervals)
☐	☒	For null hypothesis testing, the test statistic (e.g. *F*, *t*, *r*) with confidence intervals, effect sizes, degrees of freedom and *P* value noted<br>*Give P values as exact values whenever suitable.*
☒	☐	For Bayesian analysis, information on the choice of priors and Markov chain Monte Carlo settings
☒	☐	For hierarchical and complex designs, identification of the appropriate level for tests and full reporting of outcomes
☐	☒	Estimates of effect sizes (e.g. Cohen's *d*, Pearson's *r*), indicating how they were calculated

Our web collection on statistics for biologists contains articles on many of the points above.

Software and code

Policy information about availability of computer code

Data collection	QuantStudio™ Real-Time PCR Software for Q-PCR data collection.<br>Luminescence assay data for ROS production was collected by i-control 1.9 software (for infinite F200 reader, Tecan)<br>Agilent Mass Hunter Quantitative Analysis Version B.07.00 (HPLC/MS)
Data analysis	GraphPad prism7 (graphing) IBM SPSS statistics 20 (statistical analysis) FV10-ASW 3.1 Viewer (fluorescence image analysis) NTAnalysis 1.5.41 (MST assay data analysis)

For manuscripts utilizing custom algorithms or software that are central to the research but not yet described in published literature, software must be made available to editors/reviewers. We strongly encourage code deposition in a community repository (e.g. GitHub). See the Nature Research guidelines for submitting code & software for further information.

Data

Policy information about availability of data

All manuscripts must include a data availability statement. This statement should provide the following information, where applicable:

- Accession codes, unique identifiers, or web links for publicly available datasets
- A list of figures that have associated raw data
- A description of any restrictions on data availability

The data that support the findings of this study are available from the corresponding author upon reasonable request.

Field-specific reporting

Please select the one below that is the best fit for your research. If you are not sure, read the appropriate sections before making your selection.

Life sciences study design

All studies must disclose on these points even when the disclosure is negative.

Sample size	Sample sizes were determined based on pre-experiment or previous studies to reach a statistical significance (p<0.05). Specifically, sample size for ROS assay, usually n>=5, were chosen as they are common for experiments of that type. Sample size for qPCR (n=3), stomatal assay (n>=30), and Luc activity assay (n=3) were chosen based published methods. The sample for colonization assay n>=6 were based on published Bacillus colonization rates in Arabidopsis. Related references were added in Methods section. The sample for colonization assay on Amorphophallus konjac were set as n=5.
Data exclusions	One in three technical replications of several data points in Supplementary Fig. 9A were excluded as the signal intensity was obviously abnormal. Otherwise, no data was excluded from other analyses.
Replication	All attempts at replications to different independent samples were successful.
Randomization	No randomization protocols or blinded analysis were employed. Samples were randomly allocated into experiment groups.
Blinding	No randomization protocols or blinded analysis were employed. Samples were randomly allocated into experiment groups.

Reporting for specific materials, systems and methods

We require information from authors about some types of materials, experimental systems and methods used in many studies. Here, indicate whether each material, system or method listed is relevant to your study. If you are not sure if a list item applies to your research, read the appropriate section before selecting a response.

Materials & experimental systems

n/a	Involved in the study	
☐	☒	Antibodies
☒	☐	Eukaryotic cell lines
☒	☐	Palaeontology
☒	☐	Animals and other organisms
☒	☐	Human research participants
☒	☐	Clinical data

Methods

n/a	Involved in the study	
☒	☐	ChIP-seq
☒	☐	Flow cytometry
☒	☐	MRI-based neuroimaging

Antibodies

Antibodies used	His-tag mouse monoclonal antibody, Signalway Antibody, catalog number #T505, lot: AF1403.<br>Goat anti-Mouse IgG HRP conjugated secondary antibody, Signalway Antibody, #L3032-2, lot: 8715.
Validation	His-tag mouse monoclonal antibody, 1/5000 dilution for western blot, TaoChen, Jingjie Li et al, et al,PKCε phosphorylates MIIP and promotes colorectal cancer metastasis through inhibition of RelA deacetylation., Nature Communications, 8(1):939. doi: 10.1038/s41467-017-01024-2. (2017 Oct 16), PMID: 29038521. Goat anti-Mouse IgG HRP conjugated secondary antibody, 1/5000 dilution for western blot, Lei Wu, Ya-nan Jiang, Qian Tang et al,Development of an Aeromonas hydrophila recombinant extracellular protease vaccine., Microbial Pathogenesis., 53:183-188 (2012), PMID: 22874879.
